# Supplementary material for: A Likelihood Approach for Real-Time Calibration of Stochastic Compartmental Epidemic Models
Source: PLoS Comput Biol. 2017 Jan 17;13(1):e1005257. doi: 10.1371/journal.pcbi.1005257 (PMC5240920; doi:10.1371/journal.pcbi.1005257)
Supplement: S1 File — (TAR.GZ) [file pcbi.1005257.s014.tar.gz › HSPH_Online-SI-Revision/IPoi09/n100-extreme/n100-extreme_BENCH_table4.pdf]

1	R0	{2.76052, 16.3913}	{2.96947, 15.963}	{{2.96947, 15.963}, {2.96947, 15.963}}	{7.56923, 2.61297}	inf	11577.	11358.	{6203., 20482.}	34.155	0.146946	newly	4336.	4362.89	{2335., 8009.}	37.0741	0.15375	Reff	0.335762	0.318528	{0.229867, 0.437937}	31.6898	ar	92090.	93829.	{93704., 93974.}	1.88835
2	R0	{2.18202, 15.1734}	{2.93978, 19.9817}	{{2.93978, 19.9817}, {2.93978, 19.9817}}	{34.7275, 31.689}	inf	9523.	7631.56	{3968., 13084.}	45.4065	0.230711	newly	3796.	2371.75	{1221., 4091.}	53.1219	0.318263	Reff	0.491915	0.260923	{0.216603, 0.317114}	48.7494	ar	84264.	93627.3	{93451., 93834.}	11.1118
3	R0	{2.57891, 12.5971}	{2.99911, 15.6207}	{{2.99911, 15.6207}, {2.99911, 15.6207}}	{16.2937, 24.0023}	inf	6810.	9931.4	{5163., 17306.}	57.4329	0.171195	newly	3181.	3825.29	{1984., 6767.}	45.2069	0.156747	Reff	0.320791	0.279456	{0.214826, 0.37102}	28.4175	ar	90302.	94013.6	{93846., 94150.}	4.11026
4	R0	{2.5856, 9.28508}	{2.93653, 10.872}	{{2.93653, 10.872}, {2.93653, 10.872}}	{13.5725, 17.0911}	inf	4198.	7883.15	{4271., 13838.}	90.4779	0.234583	newly	2428.	4057.72	{2110., 7093.}	72.9312	0.195613	Reff	0.287622	0.261374	{0.218713, 0.331358}	19.4165	ar	90312.	93586.3	{93426., 93765.}	3.62556
5	R0	{2.4406, 12.1341}	{2.99911, 15.6207}	{{2.99911, 15.6207}, {2.99911, 15.6207}}	{22.8841, 28.7339}	inf	8911.	13170.9	{7358., 22520.}	56.0842	0.172039	newly	4440.	5172.58	{2817., 8955.}	39.947	0.145612	Reff	0.386079	0.337431	{0.233871, 0.488525}	35.618	ar	88597.	94013.6	{93846., 94150.}	6.1138
6	R0	{2.72155, 15.4094}	{2.8939, 16.8013}	{{2.8939, 16.8013}, {2.8939, 16.8013}}	{6.33279, 9.0328}	inf	11194.	10450.9	{5524., 18869.}	38.5962	0.175148	newly	4357.	3810.	{2008., 7154.}	41.0017	0.196669	Reff	0.34629	0.307441	{0.242596, 0.414754}	26.4772	ar	91682.	93276.2	{93112., 93387.}	1.73887
7	R0	{2.63781, 14.3935}	{2.96978, 18.8857}	{{2.96978, 18.8857}, {2.96978, 18.8857}}	{12.5851, 31.2099}	inf	9306.	16486.1	{9811., 27631.}	77.94	0.226483	newly	3953.	5584.92	{3210., 9662.}	49.6042	0.159266	Reff	0.327906	0.444635	{0.266894, 0.689256}	46.3533	ar	91192.	93825.4	{93652., 93970.}	2.88776
8	R0	{2.82391, 16.104}	{2.8939, 16.8013}	{{2.8939, 16.8013}, {2.8939, 16.8013}}	{2.47848, 4.32998}	inf	11648.	13434.7	{7513., 23587.}	36.6025	0.136132	newly	4404.	4958.9	{2668., 8912.}	38.0693	0.144181	Reff	0.322067	0.364598	{0.262911, 0.536124}	27.7613	ar	92664.	93276.2	{93112., 93387.}	0.660698
9	R0	{2.80645, 14.6457}	{2.8939, 16.8013}	{{2.8939, 16.8013}, {2.8939, 16.8013}}	{3.11604, 14.7183}	inf	11375.	16686.4	{10283., 25758.}	50.9076	0.163361	newly	4649.	6275.62	{3859., 10869.}	44.0052	0.145377	Reff	0.325885	0.452107	{0.29376, 0.732301}	44.0604	ar	92567.	93276.2	{93112., 93387.}	0.76618
10	R0	{1.86213, 5.4271}	{2.93597, 11.6547}	{{2.93597, 11.6547}, {2.93597, 11.6547}}	{57.6673, 114.75}	inf	1169.	7795.36	{4264., 14468.}	566.84	0.78	newly	1101.	3790.05	{2034., 7133.}	244.237	0.489818	Reff	0.480541	0.25845	{0.22099, 0.336961}	46.5158	ar	75362.	93584.1	{93441., 93733.}	24.1794
11	R0	{2.27349, 14.6338}	{2.96978, 18.8857}	{{2.96978, 18.8857}, {2.96978, 18.8857}}	{30.6265, 29.0553}	inf	9305.	13594.4	{7445., 23779.}	54.4501	0.167375	newly	3974.	4544.94	{2427., 8122.}	40.2942	0.149758	Reff	0.44615	0.364653	{0.243967, 0.519088}	42.1415	ar	85805.	93825.4	{93652., 93970.}	9.34724
12	R0	{2.77261, 14.1894}	{2.8833, 17.1847}	{{2.8833, 17.1847}, {2.8833, 17.1847}}	{3.99227, 21.1094}	inf	10089.	14866.2	{8655., 25219.}	53.3683	0.169679	newly	4154.	5449.66	{3048., 9469.}	43.4134	0.146056	Reff	0.306207	0.411702	{0.272501, 0.615325}	39.795	ar	92508.	93197.4	{93009., 93386.}	0.745208
13	R0	{2.65402, 19.3935}	{2.93978, 19.9817}	{{2.93978, 19.9817}, {2.93978, 19.9817}}	{10.7671, 3.03297}	inf	15461.	11947.	{6886., 20407.}	38.8748	0.206762	newly	4998.	3768.23	{2085., 6516.}	41.9696	0.225809	Reff	0.43088	0.33341	{0.239239, 0.445112}	40.1478	ar	91184.	93627.3	{93451., 93834.}	2.6795
14	R0	{1.75297, 4.45354}	{2.99911, 15.6207}	{{2.99911, 15.6207}, {2.99911, 15.6207}}	{71.0874, 250.748}	inf	1073.	16791.2	{10174., 26755.}	1464.89	1.17012	newly	1153.	6696.69	{3867., 11179.}	480.806	0.732592	Reff	0.522297	0.429695	{0.263862, 0.681668}	44.5175	ar	71263.	94013.6	{93846., 94150.}	31.9249
15	R0	{1.99928, 7.1501}	{2.95209, 14.037}	{{2.95209, 14.037}, {2.95209, 14.037}}	{47.6577, 96.3189}	inf	2594.	10920.3	{6449., 19791.}	320.984	0.582152	newly	1932.	4633.61	{2643., 8455.}	139.835	0.332695	Reff	0.446419	0.30049	{0.232536, 0.413676}	39.1913	ar	79602.	93710.9	{93555., 93857.}	17.7243
16	R0	{2.67398, 10.3134}	{2.86001, 13.5542}	{{2.86001, 13.5542}, {2.86001, 13.5542}}	{6.95705, 31.4232}	inf	4744.	8055.79	{3986., 12623.}	77.0157	0.207009	newly	2584.	3507.1	{1659., 5541.}	54.9999	0.170801	Reff	0.275794	0.281902	{0.229001, 0.322924}	17.1125	ar	91289.	92998.7	{92780., 93186.}	1.87281
17	R0	{2.44634, 16.3226}	{2.93978, 19.9817}	{{2.93978, 19.9817}, {2.93978, 19.9817}}	{20.1705, 22.4174}	inf	11754.	17255.9	{11429., 24300.}	49.6891	0.161692	newly	4470.	5572.89	{3524., 8114.}	36.725	0.131767	Reff	0.431632	0.476888	{0.287716, 0.732946}	40.8357	ar	88710.	93627.3	{93451., 93834.}	5.54309
18	R0	{2.02168, 10.1676}	{2.97157, 19.3547}	{{2.97157, 19.3547}, {2.97157, 19.3547}}	{46.9852, 90.3566}	inf	6854.	17815.3	{10829., 26177.}	159.926	0.393562	newly	4020.	5928.95	{3499., 9374.}	52.6404	0.167256	Reff	0.515609	0.482094	{0.281437, 0.75888}	44.9654	ar	80299.	93849.1	{93703., 93993.}	16.8745
19	R0	{2.89969, 15.009}	{2.8939, 16.8013}	{{2.8939, 16.8013}, {2.8939, 16.8013}}	{0.199677, 11.9415}	inf	11247.	16686.4	{10283., 25758.}	52.2892	0.166825	newly	4512.	6275.62	{3859., 10869.}	46.7544	0.151424	Reff	0.292405	0.452107	{0.29376, 0.732301}	55.735	ar	93349.	93276.2	{93112., 93387.}	0.105738
20	R0	{2.68933, 7.34928}	{2.93653, 10.872}	{{2.93653, 10.872}, {2.93653, 10.872}}	{9.19188, 47.9329}	inf	2277.	7883.15	{4271., 13838.}	246.208	0.487492	newly	1542.	4057.72	{2110., 7093.}	163.147	0.363985	Reff	0.246316	0.261374	{0.218713, 0.331358}	14.7404	ar	91574.	93586.3	{93426., 93765.}	2.19747
21	R0	{2.15352, 9.28074}	{2.86001, 13.5542}	{{2.86001, 13.5542}, {2.86001, 13.5542}}	{32.8063, 46.0465}	inf	5169.	11178.3	{5917., 17434.}	116.412	0.29075	newly	3172.	4929.11	{2527., 7571.}	64.5081	0.185434	Reff	0.421315	0.33357	{0.246018, 0.401631}	34.4237	ar	83845.	92998.7	{92780., 93186.}	10.9174
22	R0	{2.21953, 9.04387}	{2.9884, 12.8326}	{{2.9884, 12.8326}, {2.9884, 12.8326}}	{34.6411, 41.8928}	inf	4637.	8401.02	{4831., 13395.}	83.5652	0.225337	newly	2827.	3791.1	{2115., 6056.}	47.7903	0.148628	Reff	0.397451	0.256968	{0.215015, 0.305146}	39.2118	ar	84856.	93983.5	{93851., 94123.}	10.7565
23	R0	{2.21987, 14.5347}	{2.93978, 19.9817}	{{2.93978, 19.9817}, {2.93978, 19.9817}}	{32.4303, 37.4758}	inf	9287.	11947.	{6886., 20407.}	45.0007	0.150547	newly	3912.	3768.23	{2085., 6516.}	37.3118	0.164108	Reff	0.465951	0.33341	{0.239239, 0.445112}	43.0719	ar	85067.	93627.3	{93451., 93834.}	10.063
24	R0	{2.84268, 17.9659}	{2.7811, 19.6712}	{{2.7811, 19.6712}, {2.7811, 19.6712}}	{2.16627, 9.49187}	inf	14057.	13789.8	{8863., 20597.}	26.9971	0.121087	newly	4888.	4496.25	{2769., 6985.}	29.7596	0.140599	Reff	0.351242	0.429537	{0.293489, 0.531579}	33.8404	ar	92836.	92343.4	{92163., 92489.}	0.530631
25	R0	{2.52706, 10.3679}	{2.8833, 17.1847}	{{2.8833, 17.1847}, {2.8833, 17.1847}}	{14.097, 65.7491}	inf	5201.	14866.2	{8655., 25219.}	185.834	0.427844	newly	2743.	5449.66	{3048., 9469.}	99.4161	0.267106	Reff	0.316085	0.411702	{0.272501, 0.615325}	37.7703	ar	89623.	93197.4	{93009., 93386.}	3.98824
26	R0	{2.62835, 19.1088}	{2.93978, 19.9817}	{{2.93978, 19.9817}, {2.93978, 19.9817}}	{11.8489, 4.56805}	inf	14411.	14493.2	{8921., 23968.}	29.8193	0.129421	newly	4701.	4616.56	{2738., 7744.}	32.4043	0.142367	Reff	0.418354	0.394275	{0.257584, 0.559176}	38.4041	ar	90908.	93627.3	{93451., 93834.}	2.99124
27	R0	{2.76831, 19.4155}	{2.8833, 17.1847}	{{2.8833, 17.1847}, {2.8833, 17.1847}}	{4.1538, 11.4898}	inf	12467.	7012.05	{3448., 12673.}	53.3687	0.342675	newly	3937.	2480.3	{1201., 4464.}	51.8806	0.311865	Reff	0.347063	0.262758	{0.224609, 0.317509}	29.9402	ar	92175.	93197.4	{93009., 93386.}	1.10917
28	R0	{2.96609, 16.8903}	{2.95209, 14.037}	{{2.95209, 14.037}, {2.95209, 14.037}}	{0.472002, 16.8931}	inf	11510.	7771.35	{4342., 14417.}	47.1949	0.270323	newly	4186.	3253.58	{1806., 5989.}	44.5055	0.231564	Reff	0.274037	0.256662	{0.216034, 0.322722}	18.4493	ar	94024.	93710.9	{93555., 93857.}	0.332997
29	R0	{2.17046, 14.39}	{2.76795, 19.6041}	{{2.76795, 19.6041}, {2.76795, 19.6041}}	{27.5283, 36.2342}	inf	10054.	17154.2	{11169., 23998.}	71.2048	0.216921	newly	4398.	5663.95	{3553., 8324.}	38.2331	0.134326	Reff	0.504914	0.531217	{0.323933, 0.759747}	38.07	ar	84001.	92211.6	{92022., 92408.}	9.7744
30	R0	{2.88772, 12.4647}	{2.93653, 10.872}	{{2.93653, 10.872}, {2.93653, 10.872}}	{1.69026, 12.7777}	inf	8175.	4929.7	{2593., 8809.}	51.0475	0.315995	newly	3775.	2511.43	{1273., 4569.}	49.0613	0.287261	Reff	0.263591	0.228947	{0.205293, 0.265844}	17.1165	ar	93273.	93586.3	{93426., 93765.}	0.339142
31	R0	{2.46831, 8.14308}	{2.98388, 9.60029}	{{2.98388, 9.60029}, {2.98388, 9.60029}}	{20.8876, 17.8951}	inf	3768.	4898.45	{2711., 7871.}	46.6312	0.145203	newly	2494.	2734.71	{1467., 4458.}	40.1557	0.147071	Reff	0.317474	0.219218	{0.196697, 0.240023}	32.011	ar	88762.	93948.	{93795., 94112.}	5.84257
32	R0	{1.93236, 7.38987}	{2.79531, 12.1249}	{{2.79531, 12.1249}, {2.79531, 12.1249}}	{44.6578, 64.0746}	inf	3204.	9094.77	{5080., 13219.}	183.857	0.410379	newly	2353.	4364.77	{2349., 6239.}	88.2363	0.233672	Reff	0.482588	0.308841	{0.253087, 0.354557}	39.6852	ar	77654.	92457.4	{92317., 92603.}	19.0632
33	R0	{2.82251, 17.6954}	{2.99911, 15.6207}	{{2.99911, 15.6207}, {2.99911, 15.6207}}	{6.25684, 11.7245}	inf	15003.	9931.4	{5163., 17306.}	46.8998	0.274865	newly	5209.	3825.29	{1984., 6767.}	45.8933	0.251087	Reff	0.365938	0.279456	{0.214826, 0.37102}	34.1761	ar	92745.	94013.6	{93846., 94150.}	1.36789
34	R0	{1.87083, 12.3994}	{2.76795, 19.6041}	{{2.76795, 19.6041}, {2.76795, 19.6041}}	{47.953, 58.1052}	inf	8229.	14619.4	{8764., 22539.}	78.8698	0.227643	newly	4286.	4780.26	{2785., 7415.}	35.2264	0.138041	Reff	0.630488	0.444605	{0.293375, 0.599344}	43.8561	ar	76282.	92211.6	{92022., 92408.}	20.8825
35	R0	{2.95779, 16.3003}	{2.96947, 15.963}	{{2.96947, 15.963}, {2.96947, 15.963}}	{0.394889, 2.06929}	inf	14081.	11358.	{6203., 20482.}	37.7658	0.192851	newly	5272.	4362.89	{2335., 8009.}	39.7043	0.197384	Reff	0.314472	0.318528	{0.229867, 0.437937}	29.189	ar	93873.	93829.	{93704., 93974.}	0.0964412
36	R0	{2.18718, 14.261}	{2.96398, 17.7348}	{{2.96398, 17.7348}, {2.96398, 17.7348}}	{35.5161, 24.3587}	inf	9571.	7160.74	{3583., 12914.}	45.7204	0.246684	newly	4189.	2467.16	{1219., 4488.}	52.4584	0.327969	Reff	0.488835	0.251046	{0.208812, 0.30855}	50.8947	ar	84379.	93780.3	{93638., 93879.}	11.1417
37	R0	{2.44912, 9.31118}	{2.93597, 11.6547}	{{2.93597, 11.6547}, {2.93597, 11.6547}}	{19.8786, 25.1689}	inf	4364.	7795.36	{4264., 14468.}	80.894	0.218456	newly	2540.	3790.05	{2034., 7133.}	58.8596	0.173931	Reff	0.332493	0.25845	{0.22099, 0.336961}	26.1319	ar	88442.	93584.1	{93441., 93733.}	5.81405
38	R0	{2.05949, 5.72514}	{2.93597, 11.6547}	{{2.93597, 11.6547}, {2.93597, 11.6547}}	{42.5581, 103.571}	inf	1447.	7795.36	{4264., 14468.}	438.726	0.687346	newly	1229.	3790.05	{2034., 7133.}	208.385	0.442053	Reff	0.397749	0.25845	{0.22099, 0.336961}	36.6561	ar	81587.	93584.1	{93441., 93733.}	14.7046
39	R0	{1.75505, 8.52404}	{2.96978, 18.8857}	{{2.96978, 18.8857}, {2.96978, 18.8857}}	{69.2134, 121.558}	inf	4589.	16486.1	{9811., 27631.}	259.252	0.529928	newly	3152.	5584.92	{3210., 9662.}	78.6981	0.223696	Reff	0.605615	0.444635	{0.266894, 0.689256}	50.013	ar	70925.	93825.4	{93652., 93970.}	32.2882
40	R0	{2.24724, 13.7861}	{2.8939, 16.8013}	{{2.8939, 16.8013}, {2.8939, 16.8013}}	{28.7757, 21.8713}	inf	8230.	10450.9	{5524., 18869.}	44.8921	0.148957	newly	3630.	3810.	{2008., 7154.}	40.569	0.162687	Reff	0.451088	0.307441	{0.242596, 0.414754}	38.2388	ar	85257.	93276.2	{93112., 93387.}	9.40595
41	R0	{1.77635, 8.62406}	{2.96947, 15.963}	{{2.96947, 15.963}, {2.96947, 15.963}}	{67.1669, 85.0984}	inf	4190.	11358.	{6203., 20482.}	171.075	0.39303	newly	2830.	4362.89	{2335., 8009.}	61.0698	0.174878	Reff	0.557952	0.318528	{0.229867, 0.437937}	51.2648	ar	72941.	93829.	{93704., 93974.}	28.6368
42	R0	{1.80397, 5.4932}	{2.93597, 11.6547}	{{2.93597, 11.6547}, {2.93597, 11.6547}}	{62.7505, 112.166}	inf	1509.	5007.89	{2683., 9592.}	231.868	0.471315	newly	1451.	2413.08	{1278., 4656.}	71.6691	0.19568	Reff	0.511444	0.229014	{0.20725, 0.271519}	55.222	ar	73232.	93584.1	{93441., 93733.}	27.7912
43	R0	{2.83096, 16.7077}	{2.93978, 19.9817}	{{2.93978, 19.9817}, {2.93978, 19.9817}}	{3.84393, 19.5958}	inf	13176.	17255.9	{11429., 24300.}	37.9682	0.132366	newly	4750.	5572.89	{3524., 8114.}	33.0954	0.125204	Reff	0.342093	0.476888	{0.287716, 0.732946}	48.5238	ar	92740.	93627.3	{93451., 93834.}	0.956738
44	R0	{2.73678, 11.6021}	{2.80569, 11.6897}	{{2.80569, 11.6897}, {2.80569, 11.6897}}	{2.51792, 0.755036}	inf	5400.	4715.93	{2251., 7106.}	40.2142	0.193525	newly	2650.	2283.23	{1086., 3469.}	41.434	0.201633	Reff	0.263771	0.24986	{0.227093, 0.272152}	10.5954	ar	92003.	92572.9	{92404., 92749.}	0.619418
45	R0	{2.14235, 6.98601}	{2.99939, 13.7076}	{{2.99939, 13.7076}, {2.99939, 13.7076}}	{40.0047, 96.215}	inf	1905.	8650.06	{4938., 14309.}	354.072	0.613913	newly	1420.	3699.21	{2103., 6155.}	160.508	0.368564	Reff	0.389458	0.258393	{0.212657, 0.309507}	38.0749	ar	82974.	94036.2	{93909., 94200.}	13.3322
46	R0	{2.23409, 9.21289}	{2.99911, 15.6207}	{{2.99911, 15.6207}, {2.99911, 15.6207}}	{34.243, 69.5527}	inf	5803.	16791.2	{10174., 26755.}	189.355	0.437071	newly	3539.	6696.69	{3867., 11179.}	90.3522	0.250816	Reff	0.407766	0.429695	{0.263862, 0.681668}	38.977	ar	85163.	94013.6	{93846., 94150.}	10.3926
47	R0	{2.12258, 8.15265}	{2.93597, 11.6547}	{{2.93597, 11.6547}, {2.93597, 11.6547}}	{38.3208, 42.956}	inf	3887.	7795.36	{4264., 14468.}	100.981	0.26016	newly	2681.	3790.05	{2034., 7133.}	53.9229	0.165714	Reff	0.420441	0.25845	{0.22099, 0.336961}	39.7209	ar	82690.	93584.1	{93441., 93733.}	13.1746
48	R0	{2.71771, 10.4636}	{2.80569, 11.6897}	{{2.80569, 11.6897}, {2.80569, 11.6897}}	{3.23728, 11.7178}	inf	6665.	10706.1	{5603., 16026.}	65.9435	0.194107	newly	3585.	5304.62	{2699., 7989.}	56.2366	0.169088	Reff	0.28718	0.329723	{0.256496, 0.391843}	19.7872	ar	91716.	92572.9	{92404., 92749.}	0.934279
49	R0	{2.44369, 6.66477}	{2.98388, 9.60029}	{{2.98388, 9.60029}, {2.98388, 9.60029}}	{22.1055, 44.0453}	inf	1262.	4898.45	{2711., 7871.}	288.15	0.534998	newly	921.	2734.71	{1467., 4458.}	196.928	0.415119	Reff	0.285399	0.219218	{0.196697, 0.240023}	25.4574	ar	88827.	93948.	{93795., 94112.}	5.76512
50	R0	{2.77383, 17.9775}	{2.97157, 19.3547}	{{2.97157, 19.3547}, {2.97157, 19.3547}}	{7.12877, 7.66069}	inf	11601.	14876.4	{8267., 24078.}	41.1398	0.141128	newly	3992.	4878.31	{2610., 8636.}	40.281	0.142062	Reff	0.332083	0.393241	{0.254218, 0.56439}	37.9604	ar	92334.	93849.1	{93703., 93993.}	1.64087
